# Identification and Validation of High LD Hotspot Genomic Regions Harboring Stem Rust Resistant Genes on 1B, 2A (*Sr38*), and 7B Chromosomes in Wheat

**DOI:** 10.3389/fgene.2021.749675

**Published:** 2021-10-01

**Authors:** Shamseldeen Eltaher, Amira M. I. Mourad, P. Stephen Baenziger, Stephen Wegulo, Vikas Belamkar, Ahmed Sallam

**Affiliations:** ^1^ Department of Plant Biotechnology, Genetic Engineering and Biotechnology Research Institute (GEBRI), University of Sadat City (USC), Sadat, Egypt; ^2^ Department of Agronomy, Faculty of Agriculture, Assiut University, Assiut, Egypt; ^3^ Department of Agronomy and Horticulture, University of Nebraska–Lincoln, Lincoln, NE, United States; ^4^ Department of Plant Pathology, University of Nebraska–Lincoln, Lincoln, NE, United States; ^5^ Department of Genetics, Faculty of Agriculture, Assiut University, Assiut, Egypt

**Keywords:** Triticum aestivum L, stem rust, LD, association mapping, GBS, gene annotation, genomic region

## Abstract

Stem rust caused by *Puccinia graminis f.* sp. *tritic*i Eriks. is an important disease of common wheat globally. The production and cultivation of genetically resistant cultivars are one of the most successful and environmentally friendly ways to protect wheat against fungal pathogens. Seedling screening and genome-wide association study (GWAS) were used to determine the genetic diversity of wheat genotypes obtained on stem rust resistance loci. At the seedling stage, the reaction of the common stem rust race QFCSC in Nebraska was measured in a set of 212 genotypes from F_3:6_ lines. The results indicated that 184 genotypes (86.8%) had different degrees of resistance to this common race. While 28 genotypes (13.2%) were susceptible to stem rust. A set of 11,911 single-nucleotide polymorphism (SNP) markers was used to perform GWAS which detected 84 significant marker-trait associations (MTAs) with SNPs located on chromosomes 1B, 2A, 2B, 7B and an unknown chromosome. Promising high linkage disequilibrium (LD) genomic regions were found in all chromosomes except 2B which suggested they include candidate genes controlling stem rust resistance. Highly significant LD was found among these 59 significant SNPs on chromosome 2A and 12 significant SNPs with an unknown chromosomal position. The LD analysis between SNPs located on 2A and Sr38 gene reveal high significant LD genomic regions which was previously reported. To select the most promising stem rust resistant genotypes, a new approach was suggested based on four criteria including, phenotypic selection, number of resistant allele(s), the genetic distance among the selected parents, and number of the different resistant allele(s) in the candidate crosses. As a result, 23 genotypes were considered as the most suitable parents for crossing to produce highly resistant stem rust genotypes against the QFCSC.

## Introduction

Stem rust caused by *Puccinia graminis f.* sp. *tritici (Pgt*) Erikss and Henning, has been devastating to wheat (*Triticum aestivum* L.) through many decades of production especially during the 1950s in the United States(Leonard 2001; Leonard and Szabo 2005). In recent years, stem rust losses have been minor in the U.S. due to the successful national barberry (Berberis vulgaris L.) eradication program (the alternate host for *P. graminis*), identifying stable sources of resistance, reducing potential new races in the population, and monitoring potential new races of the pathogen through a global network (Kolmer 1996; Hartman et al., 2016). To date, more than 80 stem rust (Sr) genes have been described in tetraploid and hexaploid wheat, and their wild relatives, (online Sr gene catalog, Singh, 2017). In Nebraska and the United States, QFCSC has been reported as the predominant stem rust race (Jin 2005). Although there have been many research efforts to understand the genetic control of this race, further studies are needed to reveal major and minor genes controlling the resistance against this race (Mourad AMI. et al., 2018).

The winter wheat breeding program in the University of Nebraska-Lincoln aims to select and produce wheat cultivars having high yield attributes, winter survival, disease resistance (including stem rust) and end-use quality (Baenziger et al., 2001). In this program, thousands of crosses are made followed by phenotyping and genotyping to select the most promising genotypes for future breeding program. Although phenotyping for stem rust resistance is routinely performed for all populations each year, but the emergence of new races and weather conditions could affect the progress of selection. Therefore, a fruitful selection should be done at the phenotypic and genotypic levels. Identifying new genes controlling stem rust resistance is one of the main targets to release promising resistant winter wheat cultivars. Phenotypic selection for resistance to stem rust at seedling stage is very important as it provides more understanding about the genetic control of stem rust in the evaluated genotypes and allows pyramiding of many resistant genes by crossing the selected genotypes. This type of selection which used in our study is based on a visual score using various scales such as (Stakman et al., 1962). The problem with the visual score is that it is dependent on the precision of human scores (Sallam et al., 2015), which could lead to errors in the evaluation if many individuals involved in this process. Consequently, phenotypic selection for genotypes may be ineffective. Genotyping with known stem rust genes (such as *Sr24, Sr25, Sr26, Sr32, Sr33, Sr36, Sr37, Sr38, Sr39, Sr40, Sr43, Sr44, Sr45, Sr47, Sr50, Sr51, Sr52* and *Sr53*) is useful to confirm the phenotypic selection (Bariana and McIntosh 1993; Dundas et al., 2007; Anugrahwati et al., 2008; Qi et al., 2011; Liu et al., 2011, 2013; Niu et al., 2011, 2014; Klindworth et al., 2012; Periyannan et al., 2013; Mago et al., 2013; Mcintosh et al., 2013; Yu et al., 2017). Moreover, genotyping-by-sequencing method became one of the common methods that is involved in crop breeding and improvement because it generates hundred thousand to millions of SNPs that can be used for genome-wide association study (GWAS). The GWAS is used to detect genes associated with target traits such as stem rust resistance (Amira M. I. Mourad A. M. I. et al., 2018; Juliana et al., 2018; Kumar et al., 2020). Identification of SNP markers associated with stem rust by GWAS can lead to converting these SNPs to KASP markers which have advantages over other DNA molecular markers (Sallam et al., 2017; Kaur et al., 2020)in marker-assisted selection. Using phenotypic selection combined with GWAS results, genotyping with known stem rust genes and genetic diversity will help to accelerate breeding program by selecting the true-promising genotypes as it was suggested in wheat, rice (*Oryza sativa* L.) and barley (*Hordeum vulgare* L.) by (Abou-Zeid and Mourad 2021; Ghazy et al., 2021).

In a recent study, the plant materials (hereafter referred to as DUP 2017) represent part of the preliminary yield trial of Nebraska winter wheat breeding program that is used for releasing new wheat cultivars. The DUP2017 genotypes were evaluated for grain yield in nine U.S.A. locations and high yielding genotypes were found (Eltaher et al., 2021). Moreover, the genetic diversity and population structure were extensively studied in this population and three subpopulations were detected (Eltaher et al., 2018). In addition to the previous studies carried on these DUP2017 genotypes, breeding efforts included a precise selection for stem rust resistance in the recent study. This selection was done based on phenotyping combined with extensive and detailed genetic analyses (GWAS, genotyping with expected stem rust genes, linkage disequilibrium, genetic diversity, and population structure). The DUP2017 genotypes were derived from different crosses among parents which some possessed well known stem rust resistance genes, Sr*38* and *Sr24* genes.

The objectives of this study were to: 1) screen a nursery of 212 Nebraska winter wheat genotypes for their resistance to stem rust race QFCSC, the common race in the United States, 2) identify SNP markers associated with stem rust resistance using GWAS, 3) screen the presence of *Sr38* and *Sr24* genes in the population, 4) select the most promising stem rust resistance genotypes to be used in future breeding programs.

## Materials and Methods

### Plant Materials

A collection of 212 randomly selected genotypes from 270 F_3:6_ lines (Nebraska Duplicate Nursery, syn. DUP 2017) were selected for this study. As mentioned previously, DUP2017 is the preliminary yield trial and the lines are developed from of 800–1,000 crosses among elite Nebraska adapted and cultivars from Great Plains states (Eltaher et al., 2018). The pedigree of all 212 genotypes is presented in Supplementary Table S1.

### Stem Rust Experiment at the Seedling Stage

The reaction to stem rust race QFCSC was evaluated at the seedling stage using 212 F_3:6_ lines. In addition, four check cultivars; Morocco and “Cheyenne” as susceptible and “Jagger and Arapahoe” as stem rust resistance checks were included. Stem rust spores (race QFCSC) were previously collected from naturally infected field-grown wheat, then increased in the greenhouse using a highly susceptible cultivar (McNair 701). Two hundred and twelve genotypes and four check cultivars were evaluated in a randomized complete block design with four replicates: two at the plant pathology greenhouses, University of Nebraska Lincoln, UNL and the other two at the USDA-ARS at Kansas State University (KSU).

### Inoculation and Incubation

The evaluation was done at the seedling stage by inoculating three leaves of all the genotypes using a pressurized atomizer to uniformly spray an aqueous suspension of freshly harvested urediniospores of race QFCSC (1 mg ml^−1^ in 3 ml Soltrol 170 mineral oil) (Sigma-Aldrich Corp.) according to (Rowell and Olien 1957). Inoculated plants were kept in a dark moist chamber at 21°C with 100% relative humidity for 16 h after inoculation and then moved to a growth chamber set at 20°C/18°C and a 16/8-h light/dark cycle. The stem rust symptoms were scored on the 14th day after inoculation when the rust pustules fully erupted on the inoculated leaves (Jin et al., 2007).

### Infection Types Scored

Rust ratings of three young leaves were averaged to reflect an overall rust score of the infected plants. Genotype reaction to stem rust was determined based on infection types (ITs) using a 0–4 scale (Stakman et al., 1962; Roelfs and Martens 1987). Categorical Stakman infection types on the 0–4 scale (Stakman et al., 1962) were converted to a linearized 0–9 scale removing “+,” “−,” and “; ” notations used in the Stakman scale. The 0–4 Stakman scale corresponds to distinct categories of infection types as follows: “0” = no visible uredinia or hypersensitive flecking, “; ” = hypersensitive flecking, “1” = small, round uredinia with necrosis or chlorosis, “2” = small-to medium-sized uredinia with green islands surrounded by chlorosis, “3” = medium-sized uredinia with or without chlorosis, “4” = large uredinia without chlorosis. For plants with heterogeneous infection types, all infection types were recorded. For each infection type, ‘+’ or ‘− ‘was used to indicate size variation compared to typical infection types. Stakman ITs “0,” “; ,” “1−”, “1,” “1+,” “2−,” “2,” “2+,” “3−,” “3,” “3+,” and “4” were converted to linear values 0, 0, 1, 2, 3, 4, 5, 6, 7, 8, 9, and 9, respectively. Genotypes with ITs from 0 to three were rated as resistant, 4 to 5 as moderately resistant, and 6–9 as susceptible as described in (Kumssa et al., 2015)

### Genotyping-By-Sequencing and SNP Calling

Genomic DNA was extracted from the wheat leaves of two to three young two-week-old seedlings using BioSprint 96 DNA Plant Kits (Qiagen Valencia, California, United States) as described in (Eltaher et al., 2018). The extracted DNA were sent to USDA-ARS lab, Manhattan, KS, for genotyping-by-sequencing (GBS), simple sequence-repeat (SSR), or sequence-tagged site (STS) markers that link to known rust resistance genes. Some stem rust resistance genes were predicted in some genotypes based on their pedigrees, such as *Sr24, Sr38, Sr31*, and *Sr1RS* ^*Amigo*^, so SSR and STS markers for these genes were screened for those genotypes (Mourad et al., 2019). Also, genotyping-by-sequencing (GBS) was done as Poland *et al.* (2012), described previously. The SNPs is called with default parameters using the TASSEL v5.2.40 GBS analytics pipeline (Bradbury et al., 2007). The GBS-tags were aligned to the reference genome using Burrows-Wheeler Aligner (Li et al., 2009). The reference genome v1.0 of the “Chinese Spring” genome assembly from the International Wheat Genome Sequencing Consortium (IWGSC) was used in SNP calling. The raw sequence data of the DUP2017 genotypes of the current study along with 6,791 other genotypes previously genotyped in our program were combined for SNP calling to increase the coverage of the genome and read depth at SNP sites (Zhang et al., 2015; Hussain et al., 2017; Belamkar et al., 2018). SNPs were removed from the dataset if they were either monomorphic, showed more than 20% missing values, had conflicting calls from SNPs, or exhibited minor allele frequencies (MAF) of less than 5% (Zhang et al., 2015; Hussain et al., 2017).

### Statistical Analysis

#### The Analysis of Variance and Genetic Variations

The analysis of variance for stem rust resistance was performed with PLABSTAT software (Utz 1997). The data was analyzed using the following equation.

$$Y_{ij}=µ+g_{i}+r_{j}+ge_{ij}$$

where Yij is observation of genotype i in replicate j; µ is the general mean; g_i_, e_j_ are the main effects of genotypes and replications, respectively; ge_ij_ is genotype × replications interaction of genotype i with replicate j. The random effects were assigned to genotypes whereas the fixed effects were assigned to replications. Broad-sense heritability was calculated as

$$H^{2}=\;V_{g}/V_{P}$$

where V_g_ is the genotypic variation and V_p_ is phenotypic variation.

### Population Structure, Kinship Matrix Estimation

The population structure for the F_3:6_ Nebraska winter wheat was performed using the criteria described in (Eltaher et al., 2018). The analysis was done by STRUCTURE 3.4.0 (Pritchard et al., 2000) and the kinship matrix (K) was estimated using TASSEL v5.2.40 (Bradbury et al., 2007).

### Single Marker Analysis (SMA) and Genome-wide Association Study

Single marker analysis was performed using converted phenotypic data (0–9 scale) and genotypic data (SMA) that link to known stem rust resistance gene especially *Sr38* using STS marker VENTRIUP-LN2 and *Sr24* using STS marker Sr24#12. The SMA analysis was done using PowerMarker software V. 3.25 (Liu and Muse 2005).

GWAS for stem rust resistance was performed using 11,991 SNPs markers after filtration of minor allele frequencies (MAF<0.05) were removed and excluding all heterozygous loci which were calculated as missing values. The phenotypic means for both traits and SNPs were subjected to association analysis using a mixed linear model (MLM) in TASSEL v5.2.40 software (Bradbury et al., 2007).

Each SNP marker was then fitted into the regression equation to generate a *p*-value. Marker–trait associations were considered significant at false discovery rate (FDR) at 5% significance level. For each regression model, the SNP markers were ranked from smallest to largest *p*-values. A conservative, close approach to previous studies (Pasam et al., 2012; Gao et al., 2016; Visioni et al., 2018; Kumar et al., 2020) was considered to minimize the risk of neglecting any significantly related marker annotating the resistance of the stem rust. The phenotypic variance explained by significant makers (*R* ^2^) was determined using TASSEL v5.2.40. Manhattan plots for stem rust were visualized using FarmCPU package (Liu et al., 2016). Linkage disequilibrium (*r* ^2^) was estimated using TASSEL 5.0 between each pair of SNPs located on the same chromosome. The LD heatmap was visualized using ‘LDheatmap’ R package (Shin et al., 2006)

### Candidate Genes Linked With Stem Rust

Important markers detected in the SMA were subjected to in silico annotation. The flanking sequence of these markers was obtained from the 1 kb upstream and downstream of the SNP position2 *EnsemblPlants* database. The flanking sequence was used to make a query against IWGSC RefSeq v1.0 and v1.1 to obtain the reference physical map positions of these markers (Appels et al., 2018). Each significant SNP was selected according to its falling inside the gene models. Functional annotation of the genes harboring significant SNPs was retrieved from the genome annotations provided by IWGSC and examined for their association with stem disease resistance.

## Results

### Genetic Variation for stem Rust Resistance Trait

**FIGURE 1:**
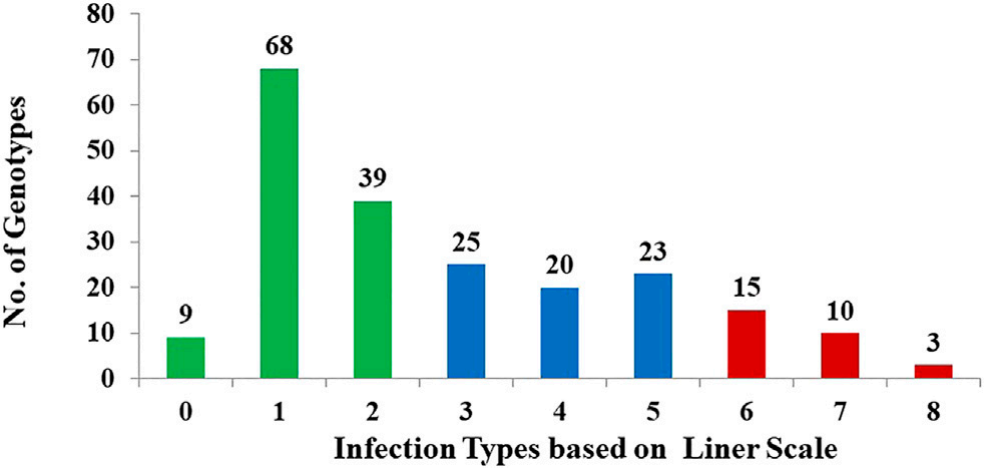
Frequency distribution of stem rust severity scores For 212 randomly selected F 3:6 genotypes to infection with the common stem rust race in Nebraska, QFCSC using the linear scale. Green bars refer to the highly resistant genotypes, blue bars refer to moderate resistance, and red bars refer to susceptible genotypes.

The analysis of variance revealed highly significant (*p* < 0.01) means squares for genotypes (G), indicating the presence of considerable differences among genotypes for resistance to stem rust (data not shown) as would be expected in a breeding program that selects for stem rust resistance (Figure 1). In the DUP 2017, the broad-sense heritability (*H* ^*2*^) for stem rust scores was 0.78. Out of the 212 genotypes, 184 (86.8%) had different degrees of resistance to this common race with a range extending from very high resistance scored as 0 (9 genotypes) to moderate resistance scored as 5 (23 genotypes). Only 28 genotypes (scored as 6 to 9, 13.2%) were susceptible to stem rust with a score ranging from susceptible with a score ranging from 5–6 (six genotypes) to highly susceptible (9) scored as 8 (two genotypes). All genotypes with a score of 0 or one were considered highly resistant to stem rust. A set of 65 highly resistant genotypes (IT < 1) were selected for further genetic investigation. The stem rust resistance of each genotype is presented in Supplementary Table S1.

### Genetic Variation in Stem Rust Resistance Based on Population Structure

**FIGURE 2:**
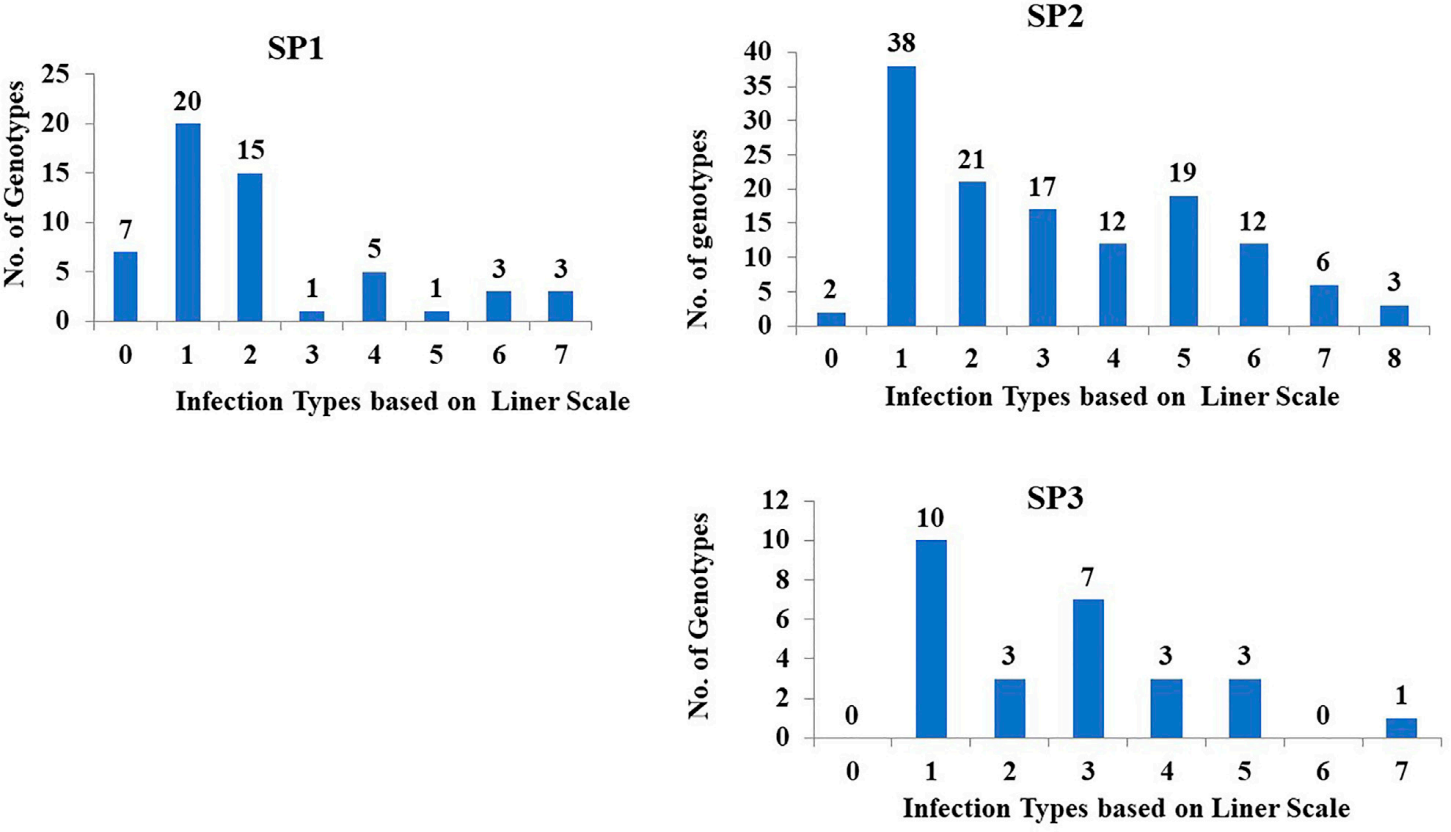
The distribution of 212 tested genotypes with the most common stem rust race in Nebraska QFCSC over the three sub-populations. SP refer to subpopulation based on population structure analysis.

Population structure (PS) analysis of DUP2017 was previously analyzed by (Eltaher et al., 2018). The results of the PS divided the genotypes into three Subgroups. The genetic variation in stem rust resistance to race QFCSC was studied in each subpopulation and presented in Figure 2. Sub-population (SP2) had the highest number of tested genotypes (130) followed by subpopulation one (SP1; 55 tested genotypes) then subpopulation three (SP3; 27 tested genotypes). In SP1, 76.4% (42 genotypes) were resistant. While 46.9% (61 genotypes) of the SP2 demonstrated resistance to stem rust. Finally, 48.1% (13 genotypes) of the SP3 displayed high degree of resistance. The mean of IT scores for genotypes in SP1 was 1.72 IT which was higher than that of those in SP2 (2.73 IT) and SP3 (2.25 IT) respectively. Single factor analysis was performed to test if there were significant differences among the three groups for stem rust resistance. The results revealed no significant differences among the three groups for the respective trait (data not shown).

### Genome-wide Association Study for Stem Rust Resistance

**FIGURE 3:**
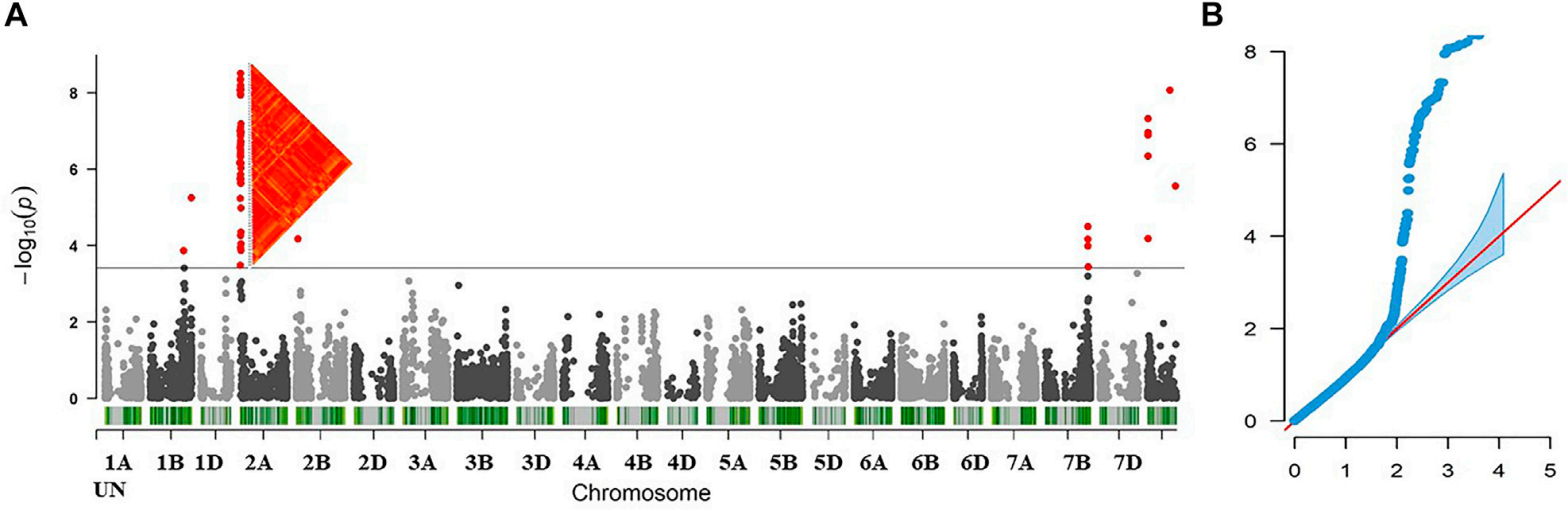
**(A)** Manhattan plot displaying SNP marker-trait association identified for stem rust resistance in GWAS using 212 wheat lines **(B)** Quantile-Quantile (QQ) plot used to evaluate the performance of the mixed linear model used for of GWAS for stem rust resistance using mixed linear model (MLM).

The mixed linear model (MLM) was used to test the genetic association between the 11,911 SNPs and the stem rust-resistance scores of all tested genotypes. The results of GWAS revealed 84 SNPs located on four chromosomes: 1B (4 SNPs), 2A (59 SNPs), 2B (1 SNP), 7B (8 SNPs) and 12 SNPs that were not mapped to a known chromosome (Unknown chromosome). The summary of GWAS results is presented in Table 1 (detailed results are presented in Supplementary Table S2). The distribution of the 84 significant SNPs across the chromosomes is illustrated *via* Manhattan plot in Figure 3A, Quantile–quantile plots of *p*-values comparing the uniform distribution of the expected−log10 *p*-value to the observed−log10 *p*-value for stem rust resistance trait showed that the MLM fitted the data well (Figure 3B).

**TABLE 1:** Summary of GWAS analysis for stem rust resistance including number of SNPs, range of *p* value, range of *R* ^2^, and range of allele effect.

Chromosome	Total no. of SNPs	–10log *p* value		PhenotypicVariation (*R* ^2^)		Allele effect range	
		Min	Max	Min	Max	Min	Max
1B	4	5.5642E-06	0.00038525	6.04	12.75	–2.15 (A and C)	–1.42 (T)
2A	59	3.086E-09	2.357E-06	13.66	25.61	–2.84 (G)	–2.09 (A)
2B	1	2.357E-06		2.357E-06		2.357E-06	
7B	8	3.175E-05	0.0003562	11.41	12.74	–1.90 (C)	–1.79 (T)
UN	12	8.5299E-09	0.000065646	9.55	22.74	–2.76 (T)	–1.95 (T)

The phenotypic variation explained by each SNP marker (*R* ^2^) ranged from 6.04% (S1B_561712520) to 25.61% (in both markers S2A_13028312 and S2A_13028321). The effects of alleles associated with decreased susceptibility to stem rust ranged from -2.83 (G) in both markers, S2A_13028312 and S2A_13028321, to -1.42 (T), S1B_547524267. Notably, the highest allele effects which decreased the stem rust symptoms were accounted for alleles found on 2A chromosome and those that belonged to the unknown chromosomal position.

**FIGURE 4:**
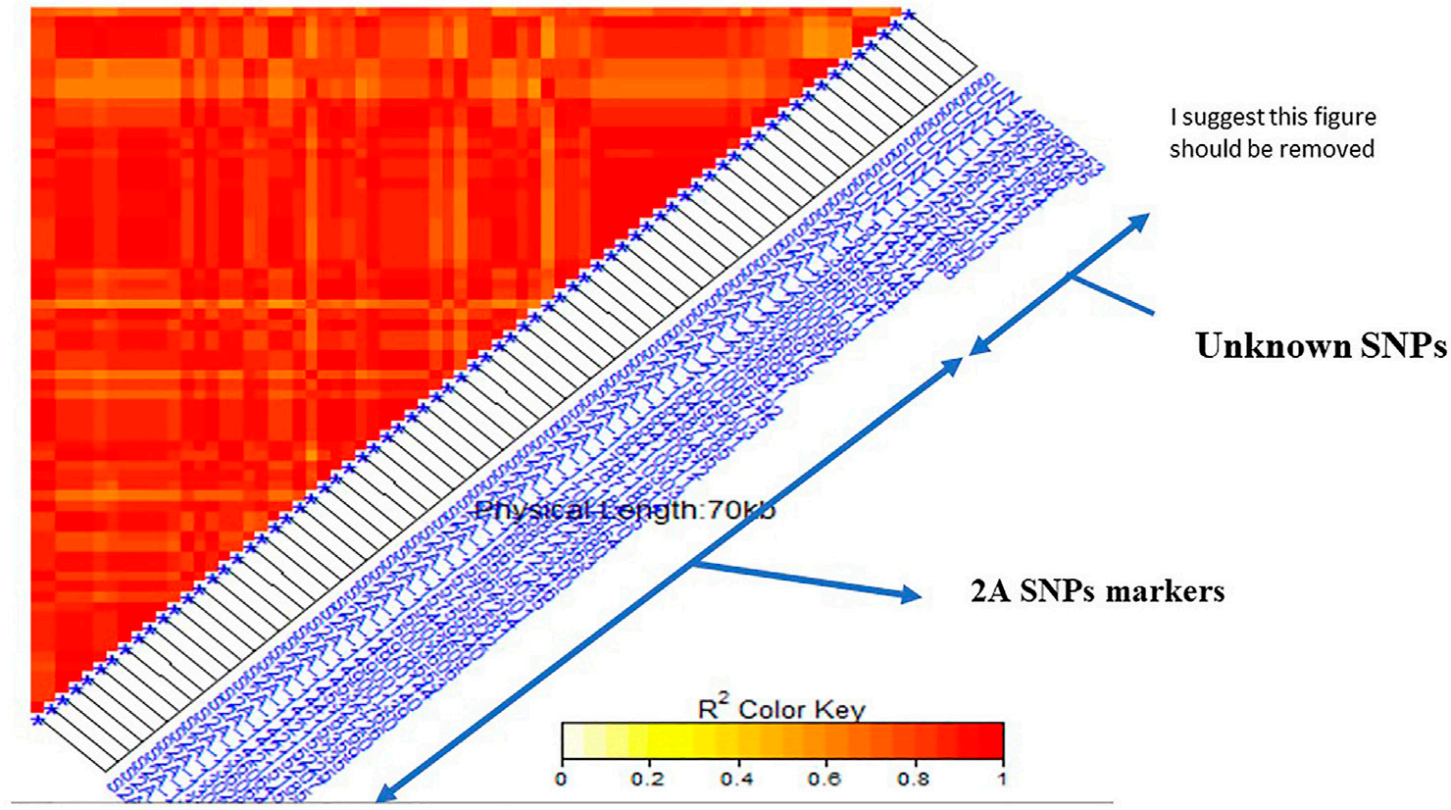
Linkage disequilibrium (LD) analysis in the tested genotypes: heatmap of LD between the 59 SNPs located on 2A and 12 Unknown SNPs markers showed highly significant LD.

The linkage disequilibrium (*r* ^*2*^) was estimated between each pair of SNPs located on the same chromosome (Supplementary Figure S1). If a group of SNPs was in significant LD, this group was named an LD genomic region (GR). A highly significant LD was found between the two SNPs located on 1B (S1B_561712520 and S1B_561712544) (GR1). Complete significant LD was found among all the SNPs located on 2A (GR2) with *r* ^*2*^ of 1. Also, the eight SNPs located on the chromosome 7B were found in a highly significant LD (GR 3). All the 12 SNPs markers located on unknown chromosomal position had significant LD indicating that they are all linked (GR 4). For unknown positional SNP markers, LD was tested between this group and SNPs located on each of the known chromosome GR to determine the most likely chromosome which the SNPs on an unknown chromosome belong to. The 12 unknown SNPs were in a high LD with the 59 SNPs located on 2A chromosome (Figure 4). According to this finding, the genomic region of the 12 SNP markers was combined with the GR2 and three major GR were identified in this study.

**FIGURE 5:**
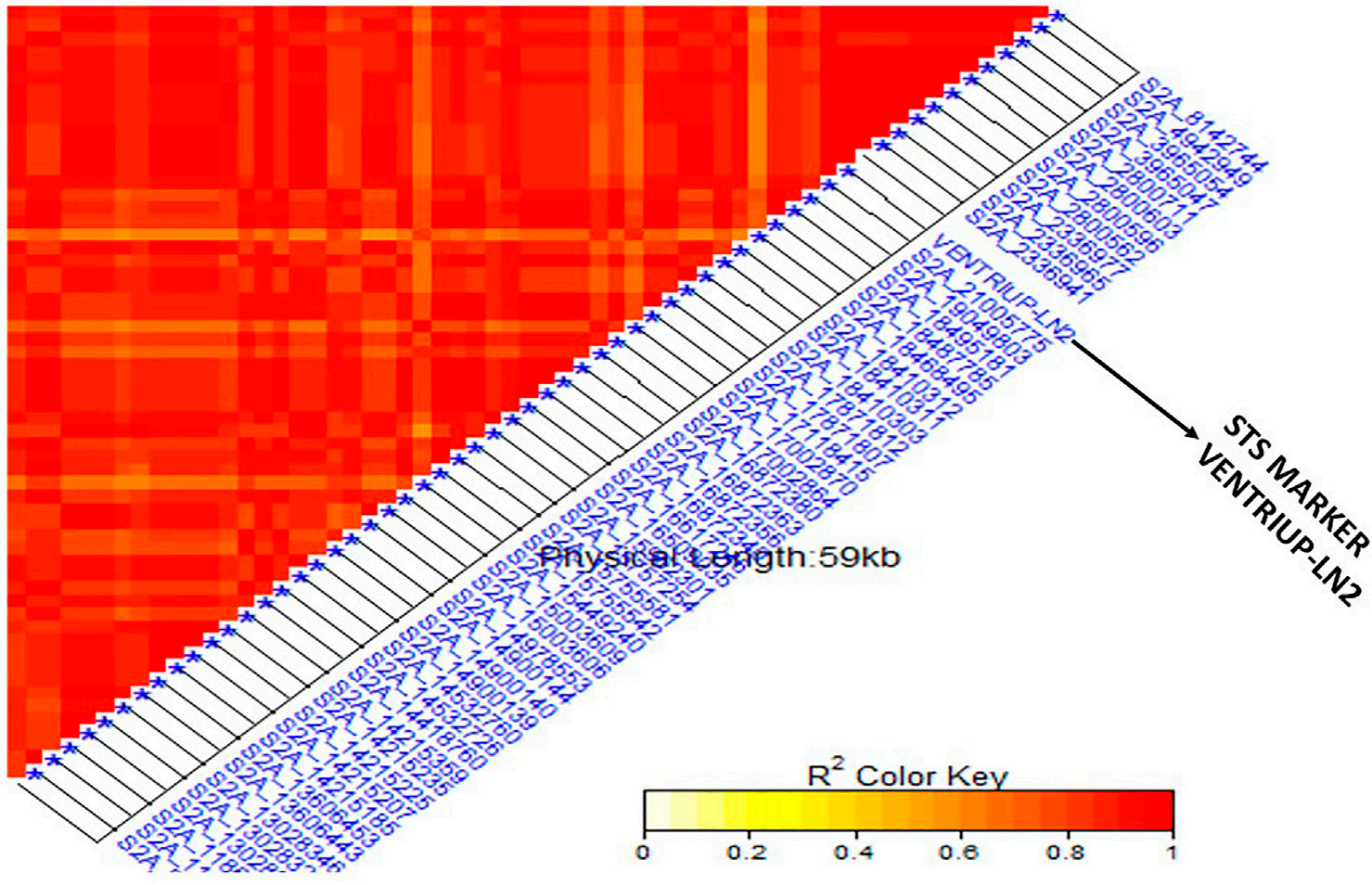
Linkage disequilibrium (LD) analysis in the tested genotypes: heatmap of LD between the specific STS marker “VENTRIUP-LN2” for Sr38 resistance gene and the 59 SNPs on 2A showed highly significant LD with the marker.

Our next step was to identify candidate genes for resistance to stem rust and to determine if the GR include known major genes for stem rust. We inspected the putative function of gene sequences corresponding to the SNPs associated with the resistant phenotype. The gene annotation analysis of the 84 SNPs markers revealed a large number of candidate genes in each genomic region (Supplementary Table S2). Many of these genes were found to be disease-related genes, particularly the hotspot (GR2) which located on the chromosome 2A. The genomic region located on chromosome 7B (GR 3) was found to be had four candidate genes (Figure 5). These gene models encoded cytochrome P450 enzymes, cytochrome P450, E-class, group I, and P-loop containing nucleoside triphosphate hydrolase.

### Single Marker Analysis for *Sr38* and *Sr24* Genes

Based on the pedigrees of the tested genotypes, some stem rust resistance genes were strongly expected such as *Sr38* (2A) and *Sr24* (3D). Based on the STS marker of the two genes (Sr24#12 and VENTRIUP-LN2), a percentage of 55.7% of the genotypes contained *Sr38* marker in their genome (118 genotypes) while only 52 genotypes with a percentage of 24.5% contained *Sr24* resistance gene (Supplementary Table S1). The results of SMA between the phenotypic data and marker data of the two genes are presented in Table 2. The SMA analysis between *Sr24* gene marker and the phenotypic data found non-significant differences between the two groups. On the other hand, a highly significant association between stem rust resistance gene *Sr38* in tested genotypes with a *p*-value of 2.45203E-33 and *R* ^2^ of 57%. The average of stem rust resistant for the group possessing the *Sr38* (118 genotypes) was 1.15 while it was 4.07 for the group not possessing the *Sr38* gene (58 genotypes). Thirty-six genotypes were heterozygous/heterogenous for the presence of *Sr38* gene. It was observed that some resistant genotypes (low stem rust score) did not have the markers associated with *Sr38* gene indicating the presence of other gene(s). The linkage disequilibrium was performed again between the STS marker VENTRIUP-LN2 (*Sr38* gene marker) and all SNPs located in GR 3 (71 SNPs) (Figure 5). The results of LD revealed a high significant LD (*r* ^*2*^ ∼ 0.90) between the SNP markers and STS marker.

**TABLE 2:** Single marker analysis for stem rust resistance in the tested genotypes explained by marker VENTRIUP-LN2 for Sr38 gene and STS marker Sr24#12 for Sr24.

	VENTRIUP-LN2	Sr24#12
Gene ID	*Sr38*	*Sr24*
*p*-value	2.45E-33	0.133472484
F-Statical	226.6559	2.269179534
F-Critical	3.895458	3.88612144
Phenotypic Variation	56.57%	1.06%
MS between group	331.3538	8.784369054
MS within group	1.461924	3.871165292
Group 1(with gene)	118^b^ (with IT average 1.15)	52 (with IT average 2.07)
Group 2(Non-gene)	58 (with IT average (4.07)	160 (with IT average 2.54)
Allele effects^a^	–1.46	–0.235

a The effect of the presence band (gene).

b There are 36 genotypes were heterozygous bands for this gene and excluded from single-marker analysis.

### Selection for the Most Promising Stem Rust Resistance Genotypes for the Upcoming Breeding Program

Three criteria were considered to determine the most promising stem rust-resistant genotypes as candidate parents for a future cross to increase stem rust resistance. These criteria were based upon the following:

First, phenotypic selection in which all highly resistant genotypes with stem rust score of 0 or one were selected. As a result, 65 genotypes were identified and included in the second stage.

Second, the presence of resistant alleles in each genotype was determined (Supplementary Table S3). The number of resistant alleles (84 marker alleles) were tested in each selected genotype to identify the genotypes which possessed the highest number of resistant alleles (Supplementary Table S3). The number of resistant alleles in the selected genotypes ranged from 11 in (NE17670) to 84 in 22 genotypes. It was noted all highly resistant genotypes possessed the GR3 and *Sr38* gene except NE17670. Therefore, it was necessary to examine the genetic distance between the NE17670 and the 22 genotypes. Consequently, the 23 genotypes were included in the third stage Third, population structure and the genetic distance matrix (GD) among the selected genotypes.

The genetic distance among the 23 genotypes is illustrated *via* dendrogram cluster analysis in Figure 7. All the 23 genotypes were from the three subpopulations (SP) according to our population structure analysis described in Eltaher et al. (2018) with 13, eight, and two for SP1, SP2, and SP3, respectively. The genetic distance extended from 0.130 (NE17627and NE17624) to 0.619 (NE17535 and NE17571). Remarkably, all the 65 genotypes identified by the first criteria had *Sr38* gene except NE17670.

## Discussion

### Genetic Variation in Stem Rust Resistance

Crop scientists face a serious challenge of increasing productivity by controlling stresses caused by biotic and abiotic effects. Stem rust of wheat, among biotic factors, poses a continuous threat through the rapid evolution of new races. Resistant cultivars are developed and considered to be the most economical and environmentally friendly tool for disease control. The primary gene pool, including indigenous collections comprising landraces, old cultivars, and breeding lines, is considered a valuable genetic resource to provide new and sustainable resistance that can be utilized for the production of today’s high yielding cultivars (Mujeeb-Kazi et al., 2013; Kumar et al., 2020; Bhavani et al., 2021). A better understanding of the genetic structure of genetic resistance is the first step towards improving and enhancing the disease resistance of this important crop. Several field and controlled greenhouse studies reported that stem rust resistance is likely under oligenic or polygenic additive regulation, due to the combined effect of multiple loci (major and minor) beneficial alleles with variable effect (Laidò et al., 2015; Saccomanno et al., 2018). Most importantly, identification of promising stem rust-resistant genotypes is the key point of the successful breeding program to truly produce high yielding cultivars with excellent resistance to stem rust.

Significant variation was observed among a collection of 212 selected genotypes from F_3:6_ lines (DUP 2017) as indicated by the analysis of variance (ANOVA). The high broad-sense heritability observed in this study indicated the reliability of data for GWAS and that selection for stem rust-resistant genotypes would be successful. The phenotypic distribution of disease response was not normally distributed which was reported also in previous studies on stem rust resistance (Gao et al., 2016; Edae et al., 2018; Saccomanno et al., 2018; Alqudaha et al., 2019; Kumar et al., 2020). In this study, more than 87% of DUP2017 genotypes were resistant to the most common stem rust race in Nebraska (QFCSC). The result was expected as selection based on resistance to stem rust is one of the main objectives for Nebraska wheat breeding program (El-basyoni et al., 2013). The genotypes studied were derived from many crosses which were known to have parents with excellent stem rust resistance. All segregating generations after crossing were subject to selection for stem rust resistance (though many plants in the field escape the disease), grain yield, and agronomic performance. Although selection for stem rust resistance was attempted in each generation, about 13% of genotypes were susceptible to QFCSC at the seedling stage. Therefore, phenotypic selection at the seedling stage alone can be misleading due to plant development and genotype × environment interaction. Phenotypic selection along with molecular genetic tools will lead to genetic improvement and a better understanding of stem rust resistance in wheat (Mourad et al., 2019; Dawood et al., 2020; Moursi et al., 2020; Ghazy et al., 2021). Moreover, selection at the seedling stage for stem rust resistance is very important as it is an efficient assay for advancing lines to the next generation.

The analysis of genetic diversity and population structure were extensively described in this population by (Eltaher et al., 2018). The genotypes were divided into three subpopulations (SP) (Supplementary Table S1). There were no significant differences in stem rust resistance among the three subpopulations (data not shown). This result indicated that the three subpopulations though genetically different were similar in their selection history and stem rust resistance. Resistant genotypes (*n* = 65) from the three subpopulations can be selected for genetic diversity and stem rust resistance for future wheat breeding (Sallam et al., 2016; Mourad et al., 2019). The three subpopulations had highly stem rust-resistant genotypes (SR scores of <1) with 25, 31 and nine genotypes from subpopulations 1, 2, and 3, respectively.

### Genome-Association Study for Stem Rust Resistance

GWAS is effective for identifying novel genes associated with stem rust resistance (Elasyoni et al., 2017; Kankwatsa et al., 2017; Edae et al., 2018; Mourad et al., 2019). In this study, we identified 84 significant MTAs distributed on different chromosomes 1B, 2A, 2B, 7B and UN (later determined to be linked to markers on 2A). A set of 78 SNPs were considered major QTLs *R* ^2^ greater than 10%. While six SNPs (S1B_547524267, S1B_561712520, S1B_561712544, S2B_28097761, SUN_12527313 and SUN_12527317) were considered a minor QTL with *R* ^2^ less than 10%. Many earlier studies reported that large-effect QTLs controlling target trait have *R* ^2^ of >10% (Hussain et al., 2017; Mourad A. M. I. et al., 2018; Alqudaha et al., 2019; Mourad et al., 2019; Niu et al., 2020). Kumar et al.)2020) detected 349 SNPs associated with stem rust resistance at seedling stage with *R* ^2^ ranging from 3.04 to 7.47% which was lower range than reported in this study. The analysis of LD between each pair of SNPs located on the same chromosome divided the 84 significant SNPs into three genomic regions. The analysis of LD provides an important information on the markers which tend to be co-inherited together from generation to generation (Sallam et al., 2016). Moreover, the analysis of LD allowed us identifying the chromosomal position of the significant SNPs with unknown chromosomal position. In the current study, we identify and validate genomic regions association with stem rust resistance. The candidate genes within each genomic region were extensively identified and described.

#### Validation of a Hot Spot Genomic Region Associated With Sr38 Gene

In our study we found a set of 59 significant SNPs located on chromosome 2A and 12 on unknown chromosome were in a highly significant LD with specific STS marker for *Sr38* stem rust gene in the VENTRIUP-LN2 translocation. The high LD found among the 12 SNPs indicated that those unknown chromosomal positions were part of the translocation that was not well mapped to the reference sequence especially in the region where the 59 SNPs were located. The LD was very useful for identifying the possible chromosomal positions of some of unknown markers. Mourad *et al.* (2019) identified a set of 17 SNPs associated with increased resistance to the same race were located on 2A and linked to *Sr38* in the DUP2015 Nebraska winter wheat. Ten of the 17 previously identified significant SNPs were common between the two studies (Supplementary Table S2) and associated with QFCSC stem rust race. The two populations (DUP2015 and DUP 2017) are genetically different and were produced from different crosses, but often had similar parents. Therefore, the 10 significant markers can be considered for marker-assisted selection. As expected, the LD between the SNP markers and *Sr38* gene confirmed the localization of *Sr38* gene in 2A chromosome. The results of the two studies recommended to use the 10 SNP markers as a strong signal for the presence of *Sr38* gene. The 10 SNPs can be converted to KASP (Kompetitive allele specific PCR) markers for further validation studies as KASP markers have more advantages over the other DNA molecular markers. While 10 markers were in common between the two studies, the other seven SNP makers in Mourad et al. (2019) were found in the raw sequence data of our study but they were excluding after marker filtration. Consequently, we can say that in the Nebraska wheat breeding program, the *Sr38* stem rust resistance gene remains a broadly used and effective resistance gene to the QFCSC local strain (Alabushev et al., 2019; Mourad et al., 2019). The other remaining SNPs (42) located on the 2A chromosome in this study were far from those detected by Mourad et al. (2019). Therefore, they could be considered novel SNPs associated with stem rust resistance.

The candidate genes in GR3 were detected and 15 gene models were identified (Supplementary Table S2). Due to the presence of many gene models in this region, we expect that this hotspot region may contains many resistance genes in addition *Sr38* gene. By looking on the stem rust genes map https://globalrust.org/knowledge-center/gene, we found that chromosome 2A containing different rust gene such as *Sr21*, *Sr32* and *Sr38/Lr37/Yr17* (Friebe et al., 1996; Nisha et al., 2015; Newcomb et al., 2016; Mourad et al., 2019). However, previous studies concluded that *Sr38*, *Sr21*, and *Sr31* are three different resistant genes which have been transferred to hexaploid wheat from different translocations (The 1973; Roelfs and McVey 1979; Bariana and McIntosh 1993; Friebe et al., 1996). Due to the presence of one GR in our results, we can conclude that *Sr21* and *Sr32* could not be the other genes expected in this genomic region and this region may carry new or unknown resistant genes. The functional annotation of the identified 15 gene models was discussed in the following paragraphs.

#### New and *VP* Putative Genomic Regions Associated With Resistance to QFCSC Stem Rust Race

Four SNPs were found to be associated with stem rust resistance on 1B chromosome. The four SNPs were found in a complete LD. S1B_547524267, which has T and C alleles, marker was found to be within *TraesCS1B02G322500* gene model which encodes pectin lyase fold/virulence factor. It was reported that pectin is among the plant cell wall components and considered an essential target for different pathogens at the early stages of infection (Wu et al., 2019). Pectin lyases are virulence factors that target the pectic components of the plant cell wall to degrade them. Therefore, this gene model is associated with increased the susceptibility to QFCSC stem rust. The allele T of S1B_547524267 marker decreased the symptoms of stem rust, while the allele C increased the stem rust symptoms. Therefore, the allele C indicate the presence of this gene. A set of QTLs at the 1B chromosome were identified using mapping populations and GWAS panel (Pozniak et al., 2008; Bhavani et al., 2011; Njau et al., 2013; Bajgain et al., 2015). Kumar et al. (2020), reported important SNP markers associated with stem rust resistance in wheat. They found 91 SNPs located on 1B chromosome. The four SNPs found in this study were far from the positions of the 92 SNPs.

For 2A chromosome 71 SNPs were found associated with stem rust resistant (59 SNPs on 2A and 12 SNPs on unknown chromosome) and around 15 gene models were recognized (Supplementary Table S2). The gene model *TraesCS2A02G003700.1* (2,336,941–2,336,977 bp) encoded to receptor like kinases (RLKs). RLKs have been discovered to play a role in both broad-spectrum, elicitor-initiated defense responses and race-specific pathogen defense as dominant resistance (R) genes. The majority of defense-related RLKs are of the leucine-rich repeat (LRR) subclass (Kruijt et al., 2004). *TraesCS2A02G010200.2* (3965047–3965054 bp) which encoded to steroidogenic acute regulatory based transfer (StART)-like domain superfamily. In insect, humans, and plants, StART proteins play a variety of roles in the transport of lipid molecules (Tang et al., 2005). These proteins consist of a modular StART domain of approximately 200 amino acids which binds and transfers the lipids. The StART domain is found in many signaling proteins and is believed to have important roles in lipid transport, lipid metabolism and cellular signaling (Soccio and Breslow 2003; Tang et al., 2005). The StART proteins plays role in the plant defense against powdery mildew in Arabidopsis by EDR2, a PH (Pleckstrin homology) and START (lipid/sterol-binding StAR-related lipid transfer) domain-containing protein (Vogel et al., 2002, 2004; Tang et al., 2005). The gene model *TraesCS2A02G028800* (13,028,312–13,028,346 bp) which encoded to F- Box domain proteins family. As one of the largest and most diverse plant gene families, F-box proteins are involved in many cellular processes, including cell cycle, circadian rhythms, embryogenesis, floral organ development, stress responses, and various signal transduction pathways. F-box proteins are reported to be related with the plant response against bacterial, viral and fungal pathogens (van den Burg et al., 2008; Piisilä et al., 2015; Li et al., 2020). Also, many of F-box proteins in higher plants have been characterized by genetic analysis and are involved in various abiotic stresses (Calderón-Villalobos et al., 2007; Xu et al., 2008; Zhang et al., 2008; Bu et al., 2014). The gene model *TraesCS2A02G028800* (13,028,312–13,028,346 bp) which encoded the ABC transporter domain superfamily. Plant ABC transporters are classified into several sub-families (ABCA - ABCH) and play diverse roles (Campbell et al., 2003). Although approximately 131 ABC transporters have been identified in Arabidopsis, via sequence similarity to known ABC transporters in other organisms, very little is known about the functions or the substrate specificities of most of these genes (Campbell et al., 2003; Jasinski et al., 2003). ABC transporters have been associated with various host-pathogen interactions. In plant pathogenic fungi, members of this transporter group play a role in providing resistance to phytoalexins (Nakaune et al., 1998; Urban et al., 1999; Schoonbeek et al., 2001; Fleissner et al., 2002; Campbell et al., 2003; Jasinski et al., 2003), and to antifungal compounds (Hayashi et al., 2002), or act as novel pathogenicity factors (Fleissner et al., 2002; Campbell et al., 2003). In addition, several gene models on chromosome 2A have been discovered to have relationships with plant protection. For example, *TraesCS2A02G042800.1, TraesCS2A02G040600.1* and *TraesCS2A02G036900.1* which encoded to Chloramphenicol acetyltransferase-like domain superfamily and Isopenicillin N synthase-like these types of proteins had antibacterial effects. The functional annotation of these gene models confirmed the presence of many stem rust resistance genes.

On 2B chromosome, one SNP (S2B_28,097,761) was found to be associated with stem rust resistance. This SNPs located very near *TraesCS2B02G057600* (28,093,392–28,097,727) which encodes to MFS transporter superfamily. The MFS transporter is a member of plant defense-related proteins that could be involved in exporting the antimicrobial compounds produced by plant pathogens, the plant-generated antimicrobial compounds; and potassium which is important during plant defense reactions (Friebe et al., 1996; Simmons et al., 2003). Antimicrobial compounds were found to provide resistance against fungal in different plants in different ways such as avoidance, enzymatic degradation, and non-degradative mechanisms(Osbourn 1999; Seybold et al., 2020). According to McIntosh atlas (McIntosh et al., 1995), many stem rust resistance genes were mapped on this chromosome such as *Sr10*, *Sr16*, *Sr9*, *Sr12*, *Sr19*, *Sr20*, *Sr23*, *Sr28*, *Sr32*, *Sr36*, *Sr39*, *Sr40*, *Sr47*, and *SrWeb*. However, more studies are definitely needed to provide more information about the significant SNP marker and resistance genes on chromosome 2B. In the study of Kumar et al. (2020), five SNPs were associated with stem rust at seedling stage with positions different from what was found in our study.

**FIGURE 6:**
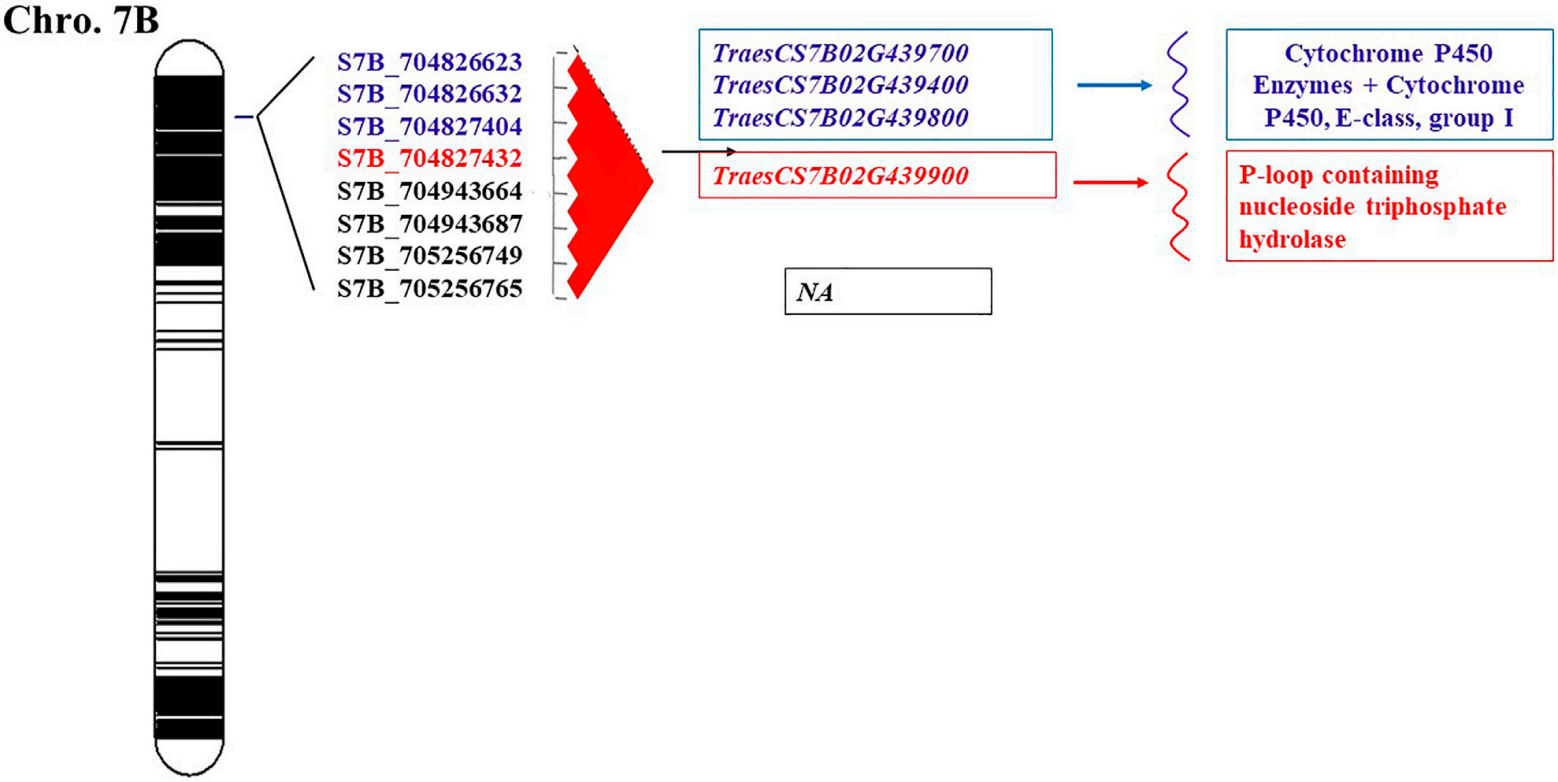
The schematic representation of genomic regions map on chromosome 7B.

A GWAS analysis showed the presence of highly significant SNPs located on 7B chromosome. This set of eight SNPs located on 7B was highly LD and considered as a genomic region (Figure 6). Previous studies on bi-parental mapping populations and GWAS panel suggested the presence of *Sr17* on 7B chromosome (Bansal et al., 2008; Yu et al., 2011, 2017; Leonova et al., 2020). However, *Sr17* is a virulent gene against QFCSC race. Therefore, the significant SNPs could not be associated with *Sr17* gene. All the four SNPs were considered major QTLs which tend to co-inherited together. The gene annotation of the candidate significant SNPs supported the results of the marker-trait association. Chromosome 7B seemed to have a very interesting genomic region including four gene models (Figure 6). Three candidate genes *TraesCS7B02G439700.1*, TraesCS7B02G439800, and *TraesCS7B02G439400.1* (704,826,623–704,827,404 pb) encoded cytochrome P450 Enzymes which play an important role in enhancing the resistance of several plant-fungal interactions including stem rust, powdery mildew (*Blumeria graminis* f. sp. *tritici*) and *Fusarium* head blight (*Fusarium graminearum)* (Becher and Wirsel 2012). S7B_704827432 was located within *TraesCS7B02G439900* gene model which encoded the protein P-loop containing nucleoside triphosphate hydrolase. This protein is encoded by many wheat disease resistance genes that are distributed across the wheat genome. The domains of this protein were found to be associated with the receptors that can detect pathogenic effectors. Interestingly, a set of 132 genes encoding P-loop containing nucleoside triphosphate hydrolase protein was found in chromosome 7B. By comparing the position of genomic region of this unidentified gene (704,826,623–705,256,765, Supplementary Table S2) with the same position of genomic region reported by Becher and Wirsel, (2012), we found that the two genes *TraesCS7B02G437400.1* (703719566) and *TraesCS7B02G441700.1* (706811897) are near the gene detected in this study. These results indicated that 7B chromosome may include very important genomic regions associated with QFCSC resistance which can be used for marker-assisted selection after validating the SNPs in a different genetic background. A set of four SNPs were associated with stem rust and located on 7B detected by Kumar et al. (2020). In this study, All SNPs located in a genomic region starting from 704,826,623 to 705,256,765 (GR3). In the study of Kumar et al. (2020), the four SNPs were located on four different positions 619883523, 638510367, 716966341 and 730906118.

Therefore, by comparing the position of SNPs and candidate genes detected in the study with the positions of SNPs and candidate genes reported by earlier studies, it can be concluded the most genomic regions detected in this study were novel. Bearing in mind that, all detected candidate genes found in this study have a strong relation with plant disease resistance in wheat.

### Utilize the Aspects of GWAS and Genetic Diversity Analysis to Identify the Most Stem Rust-Resistant Genotypes

We also expect that many stem rust resistance genes are present in our materials due to, wide range of infection types. For instance, we have 58 genotypes with non *Sr38* gene (Table 2) the average infection types ranged from (0.42–7.50). Also, the genotype (NE17670) which marked as resistance in the selected 23 genotypes with IT (0.42) and did not have SNP markers indicative of Sr38 gene, this indicated the presence of other resistance genes in our DUP2017 genotypes.

Phenotypic selection is widely used in traditional plant breeding. However, phenotypic selection could be misleading due to epistasis and the environmental or human errors which could reduce heritability and lead to ineffective selection. Selection based on genotypic and phenotypic values together can address this challenge by truly select the most promising stem rust-resistant genotypes.

**FIGURE 7:**
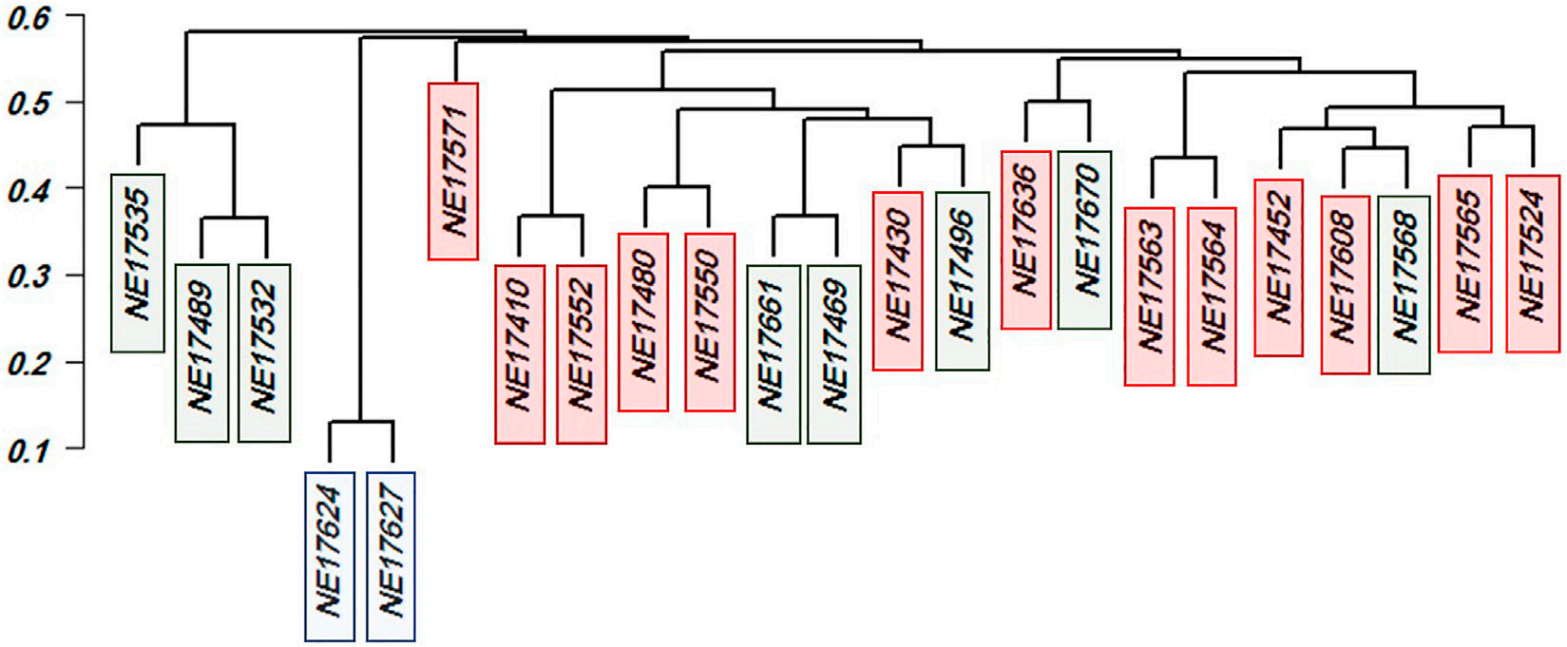
Dendrogram cluster and principal component analysis for the selecting 23 selected genotypes with the most forms of resistance infections types. Red color refer to SPI, Green color refer to SP2 and Blue color refer to SP3.

To address this challenge, genotypes were selected based on three criteria as described by (Eltaher et al., 2021). Firstly, phenotypic selection for the highest resistant stem rust genotypes. Out of the 212 genotypes, 65 highly stem rust-resistant genotypes were selected to be advanced to the next phase of selection. Secondly, the number of resistant alleles and their genomic regions, detected by GWAS, were counted in each genotype. It was very useful to identify the number of resistant alleles which each selected genotype carried as it shed the light on the number of putative genes controlling the resistance of stem rust in this population. Most of the GWAS literature overlooks the number of resistant alleles in the target genotypes. Here, the number of resistant alleles confirmed the results of phenotypic selection. For example, resistant genotypes should contain at least some of the resistant alleles detected by GWAS and the number of resistant alleles should be more than susceptible alleles. It was noted that all the selected genotypes had the SNPS for GR3 except one genotype, NE17670 which included only 11 resistant allele with an IT of 0.41. By looking to the data, we selected 22 genotypes which had the maximum number of resistant allele (84) and it was interesting to include the NE17670 genotypes as it was thought to possess other resistant genes which were not detected by GWAS. Thirdly, genetic distance and population structure among the remaining 23 genotypes was considered. Genetic distance was very useful in providing information on how each two genotypes are genetically dissimilar, hence maintain genetic diversity in the breeding program. It also provided a way to screen parents for the number of different GR. We discovered that number of GR in each genotype was not enough to select a parent. For example, NE17624 and NE17627 had 84 resistant alleles (3 GRs) against stem rust. The genetic distance based on resistant alleles was 0.13 which indicated that both genotypes are highly genetically similar. On the other hand, it was noted that NE17670 had lowest number of resistant alleles. This genotype possessed (11) resistant alleles. The genetic distance between this genotype and NE17469 was 0.62 with lines being different for 73 stem-rust related alleles. Bearing in mind that the analysis of population structure assigned NE17670 and NE17469 in SP2 (Figure 7). Therefore, NE17670 as a candidate parent should be included in the future crosses to produce cultivars having more resistance to stem rust race QFCSC. The fact that extremely genetically distant genotypes are best in the crossbreeding phase was previously described in (Eltaher et al., 2018). Consequently, hybridization between NE17670, which belongs to SP2, and any of the two genotypes (NE17624 and NE17627), which belong to SP3, should be considered, especially for pyramiding stem rust resistance genes. Hybridization of NE17670 for all 13 SP1 genotypes can also be helpful. Therefore, integration of NE17670 as a main parent in the crosses with the other genotypes in SP1, SP3 genotypes will be fruitful in producing cultivars having more resistance to the common stem rust race QFCSC on one hand, to maintain the genetic diversity among the lines on the other hand.

## Conclusion

Four LD genomic regions controlling important *Sr* genes associated with stem rust resistance were identified. In particular, the genomic region harboring Sr38, one of the most important resistant genes to stem rust, was found. The validated SNPs in this region can be converted to KASP markers which can be used for marker-assisted selection for Sr38. Moreover, important new SNPs especially those located on 7B chromosome were identified. These markers will need to be validated before using them in MAS, but the first step is completed. The gene annotation analysis revealed putative genes associated with fungal disease resistance. These results further support that GWAS was a powerful method to identify target alleles. Moreover, most of significant SNP detected by GWAS were considered with major effects in stem rust resistance except six SNPs with minor effects.

Finally, information from genetic diversity, population structure, and GWAS results were combined to identify the most promising wheat resistant genotypes as potential parents for future a breeding program. As a result, 23 genotypes were identified, and based on our results, we recommend NE17670 be used as a parent in future crosses as it may have other resistant genes which were not identified by GWAS.

## Data Availability

The datasets presented in this study can be found in online repositories. The names of the repository/repositories and accession number(s) can be found in the article/Supplementary Material. The GBS data analyzed during the current study are available in the NBCI repository, http://www.ncbi.nlm.nih.gov/bioproject/680548

## Key Message

Important genomic regions associated with resistance to QFCSC stem rust race were identified and validated. Moreover, the most promising stem rust genotypes were identified for future breeding and genetics programs.
