# Supplementary material for: Identification and Validation of High LD Hotspot Genomic Regions Harboring Stem Rust Resistant Genes on 1B, 2A (Sr38), and 7B Chromosomes in Wheat
Source: Front Genet. 2021 Oct 1;12:749675. doi: 10.3389/fgene.2021.749675 (PMC8517078; doi:10.3389/fgene.2021.749675)
Supplement: Supplementary file 1 [file Presentation1.pptx]

## Slide 1
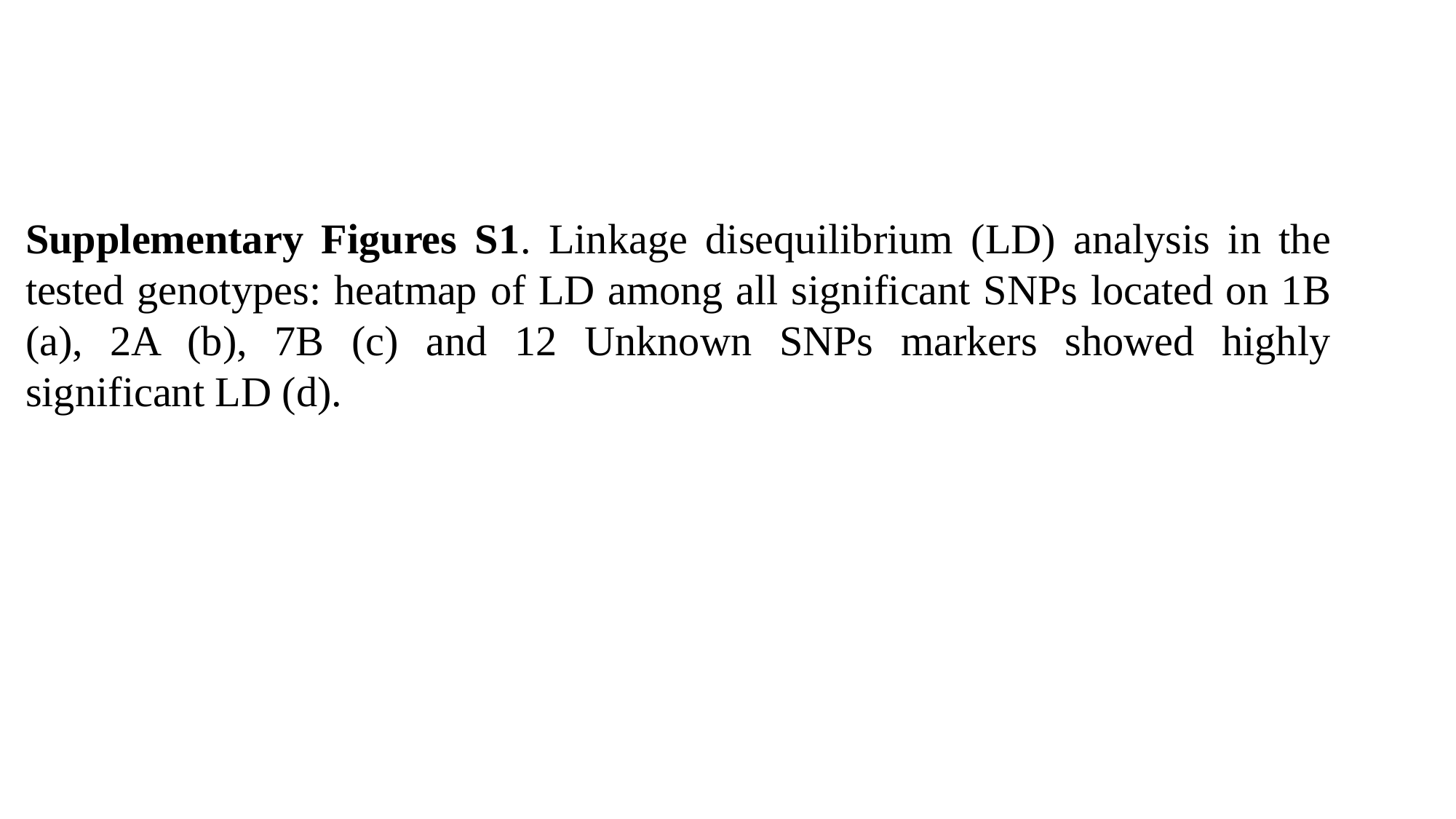

Supplementary Figures S1. Linkage disequilibrium (LD) analysis in the tested genotypes: heatmap of LD among all significant SNPs located on 1B (a), 2A (b), 7B (c) and 12 Unknown SNPs markers showed highly significant LD (d).

## Slide 2
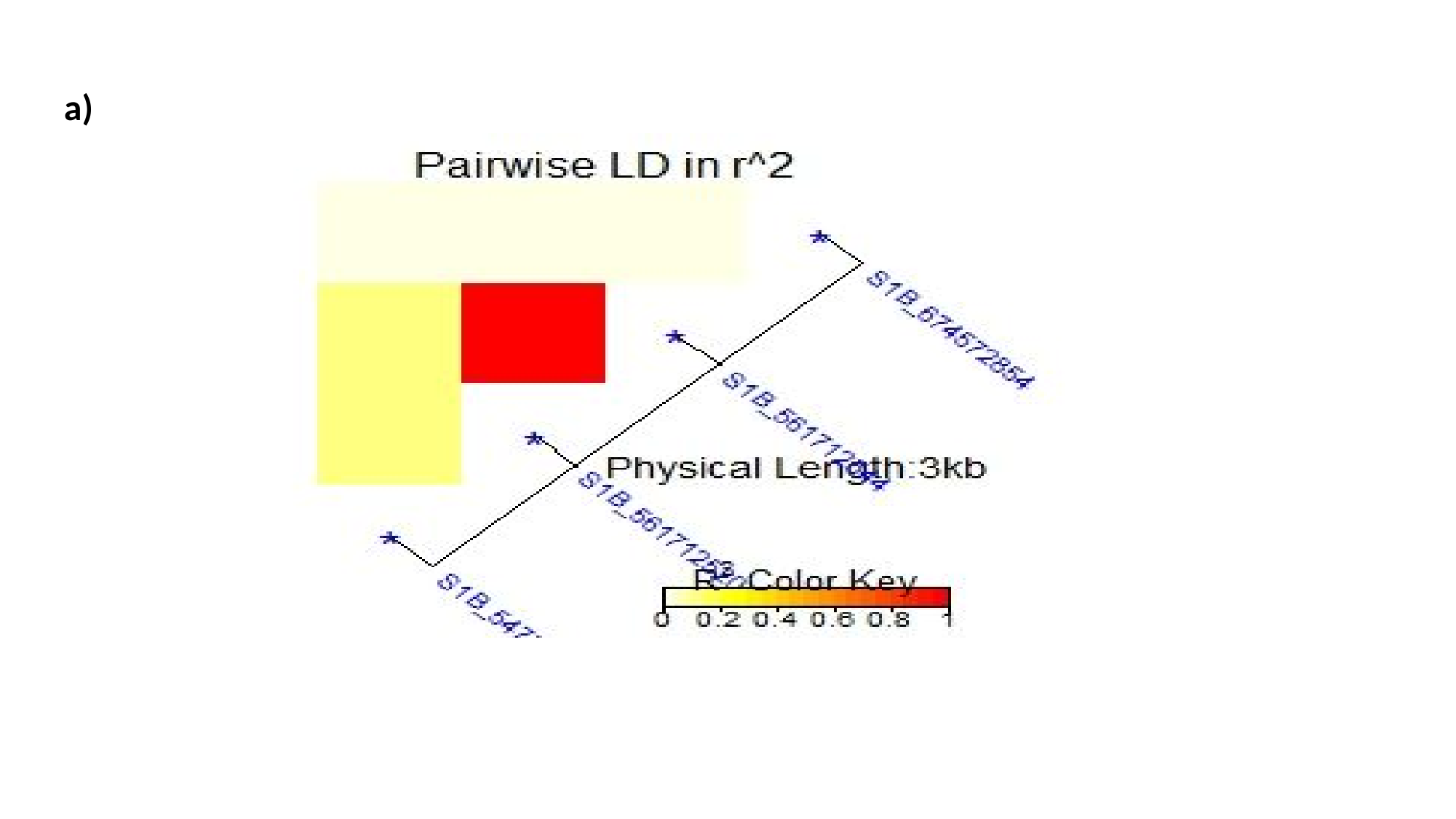

a)

## Slide 3
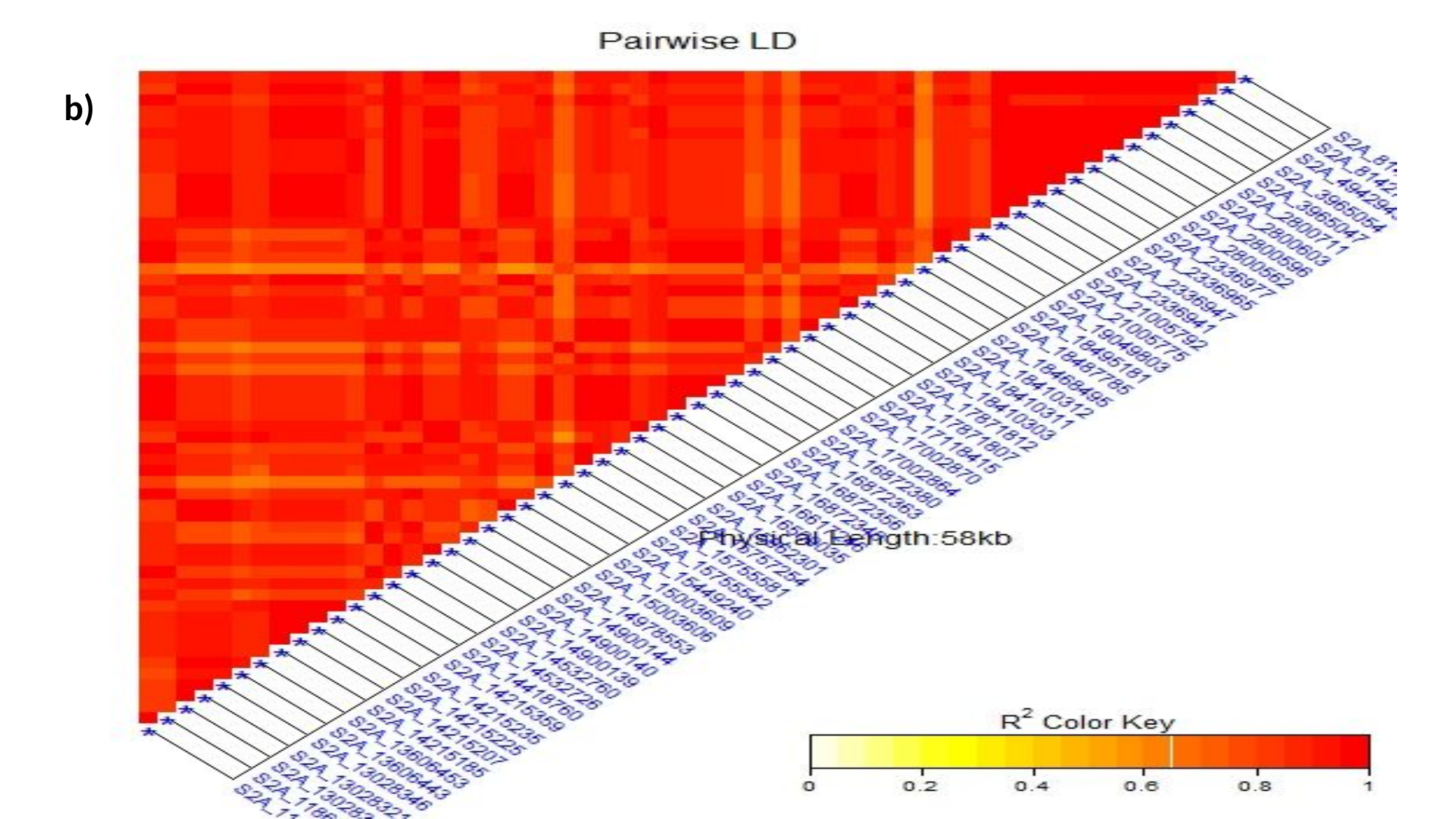

b)

## Slide 4
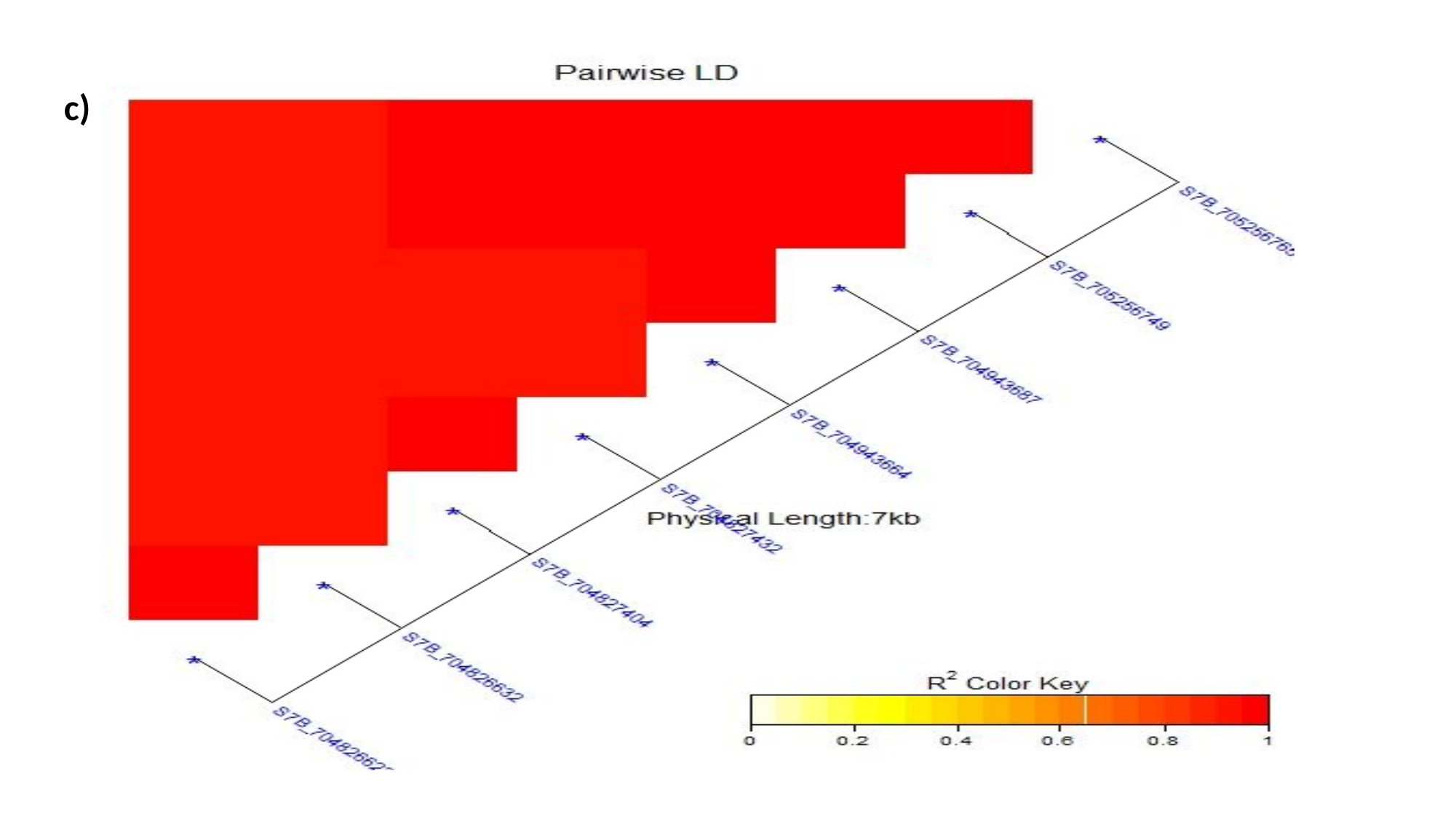

c)

## Slide 5
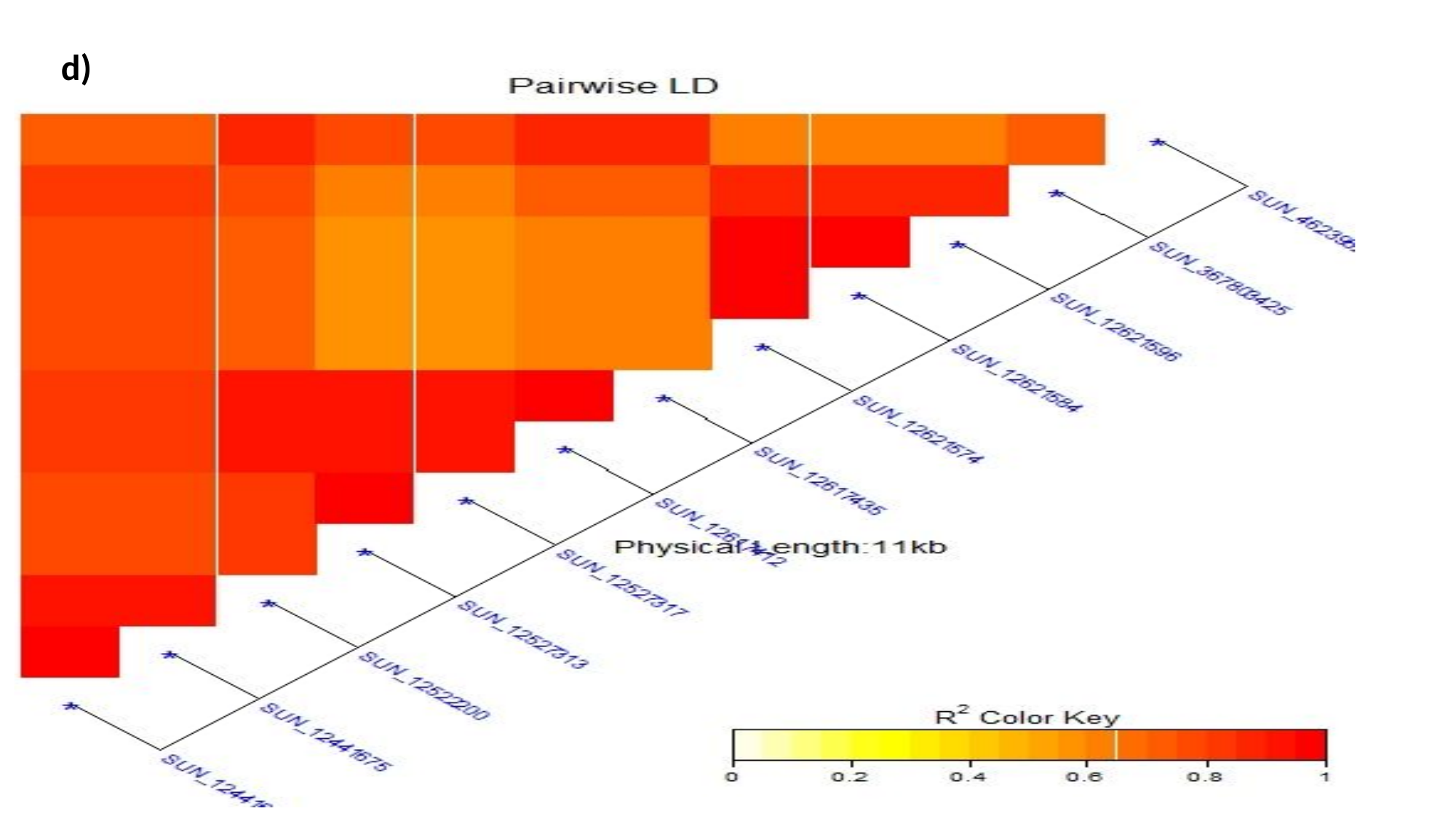

d)
